# Biomarkers of Micronutrients and Phytonutrients and Their Application in Epidemiological Studies

**DOI:** 10.3390/nu15040970

**Published:** 2023-02-15

**Authors:** Jianheng Zheng, Feng Wu, Feijie Wang, Junrui Cheng, Hong Zou, Yuan Li, Jun Du, Juntao Kan

**Affiliations:** 1 Nutrilite Health Institute, 720 Cailun Road, Shanghai 201203, China; 2 Sequanta Technologies Co., Ltd., 240 Hedan Road, Shanghai 200131, China; 3 Department of Molecular and Structural Biochemistry, North Carolina State University, Raleigh, NC 27695, USA

**Keywords:** nutritional biomarker, dietary, vitamins, minerals, phytonutrients, epidemiology

## Abstract

Nutritional biomarkers can be used as important indicators of nutritional status and play crucial roles in the prevention as well as prognosis optimization of various metabolism-related diseases. Measuring dietary with the deployment of biomarker assessments provides quantitative nutritional information that can better predict the health outcomes. With the increased availability of nutritional biomarkers and the development of assessment tools, the specificity and sensitivity of nutritional biomarkers have been greatly improved. This enables efficient disease surveillance in nutrition research. A wide range of biomarkers have been used in different types of studies, including clinical trials, observational studies, and qualitative studies, to reflect the relationship between diet and health. Through a comprehensive literature search, we reviewed the well-established nutritional biomarkers of vitamins, minerals, and phytonutrients, and their association with epidemiological studies, to better understand the role of nutrition in health and disease.

## 1. Introduction

The daily diet is closely related to human health. The nutrients from food are absorbed by the body and transformed into body tissues to maintain the full range of physiological activities and meet health needs [1,2]. It has been established that environmental cues and lifestyle choices, including dietary patterns and eating habits, may influence health and disease outcomes [3]. A healthy diet has been linked to beneficial health outcomes [4], whereas an unhealthy eating habit can increase the risks of non-transmittable chronic disease, such as cancer, obesity, cardiovascular diseases and metabolic syndrome [5,6]. Recognizing the connection between food and human metabolism is crucial for the development of efficient nutritional interventions, during which nutritional biomarkers should be defined to consider the digestibility, bio-accessibility and bioavailability of the food [7,8]. Therefore, a consensus on the selection and measurement of nutritional biomarkers is required to enable consistency in research and data interpretation [9].

Different types of biomarkers have been established in different biological matrices: blood, excretory products, noninvasive specimen, and invasive specimen. In general, biomarkers are classified into exposure biomarkers and clinical biomarkers [10,11]. Exposure biomarkers are used directly to evaluate the concentration of targeted nutrients, which reflects a nutritional status. For instance, to evaluate the total vitamin D from dietary sources and endogenic synthesis, the biomarker 25-hydroxyvitamin D [25(OH)D] in blood is widely utilized [12]. The assessment of exposure biomarkers enables the surveillance of early-stage nutrition deficiency but does not necessarily monitor the occurrence of diseases. Nevertheless, in surveillance, nutritional clinical biomarkers can be utilized as reliably quantified and assessed indicators of healthy biological processes, pathological processes, or pharmacological responses to therapeutic interventions, which can be used as indicators of hazard and population risk for disease prevention. For example, as an important biomarker of iron store status, serum ferritin could be employed as a biomarker to identify iron deficiency, which is associated with iron deficiency anemia in some undeveloped areas. WHO has set a cut-off value of serum ferritin to evaluate iron storage status to screen people who are at risk for iron deficiency so that iron supplements can be provided to the people in need [13,14].

Micronutrient and phytonutrient deficiencies have been associated with a variety of human diseases. A comprehensive report on the current state of biomarker research that evaluates the nutritional status of individual and population has been reported by an expert panel from the Biomarkers of Nutrition for Development (BOND) project. The initial phase of the BOND project involves assessing biomarkers for 6 nutrients: vitamin A, vitamin B9, vitamin B12, iodine, iron and zinc, with the aim of providing health-promoting recommendations for these nutrients [15,16,17,18,19,20]. In a parallel effort, we reviewed nutritional biomarkers of vitamins, minerals and phytonutrients and their association with health or diseases in epidemiology research, addressing the validity of these biomarkers in the context of nutrition research (Table 1).

## 2. Methods

### 2.1. Information Sources and Search Strategy

With the guidance of the keywords: “nutritional biomarker”, “epidemiology”, “vitamin biomarker”, “vitamin A”, “folate”, “vitamin B12”, “vitamin D”, “mineral biomarker”, “iodine biomarker”, “iron biomarker”, “zinc biomarkers”, and “phytonutrient biomarkers,” “polyphenol biomarkers,” “carotenoid biomarkers,” four databases—PubMed, Web of Science, Medline, and Scopus—were searched for pertinent papers from January 2017 to July 2022, related articles were gathered without regard to a specific timeframe or filter. Additional papers were found by searching the reference lists of recognized publications, including systematic reviews and meta-analyses. The research included were limited to peer-reviewed papers written in English.

### 2.2. Criteria and Study Selection

Inclusion criteria were applied following the PICOS (Participants, intervention, comparators, outcomes, and study design) model [42,43]. Articles were qualified if they were primary research studies that explored nutritional biomarkers, particularly minerals biomarkers, vitamins biomarkers, and phytonutrients biomarkers, and their associations with epidemiological investigations. These studies were extended based on the content framework of the BOND project. First, nutritional biomarkers that have been routinely used are collected, and relevant epidemiological research are needed to demonstrate the significance of nutritional biomarkers in the research. Additionally, those that contain specific details about vitamin A biomarkers, folate biomarkers, vitamin B12 biomarkers, vitamin D biomarkers, iodine biomarkers, iron biomarkers, zinc biomarkers, polyphenol biomarkers, and carotenoid biomarkers, as well as contents pertaining to the use of these biomarkers in epidemiological studies, will be included in our collection criteria. Before reviewing the complete texts of potentially eligible research, publication titles and abstracts were evaluated against the selection criteria. When there was insufficient information on the nutritional biomarker or associated epidemiological research, investigations were excluded.

### 2.3. Synthesis of Results

Two researchers analyzed the search results separately to determine if each result fulfilled the inclusion or exclusion criteria. When there was a dispute, a third researcher assessed the situation and determined whether it should be included or excluded.

## 3. Vitamin Biomarker

Vitamins are a class of trace organic chemicals that both humans and animals need to consume through diet to sustain healthy physiological processes [44]. These substances must be obtained from diet since the body cannot generate them or the amount synthesized is insufficient. The deficiency of vitamins is asymptomatic at the initial stage but may exhibit detrimental effects with an increased deficit or a prolonged period.

**Figure 1:**
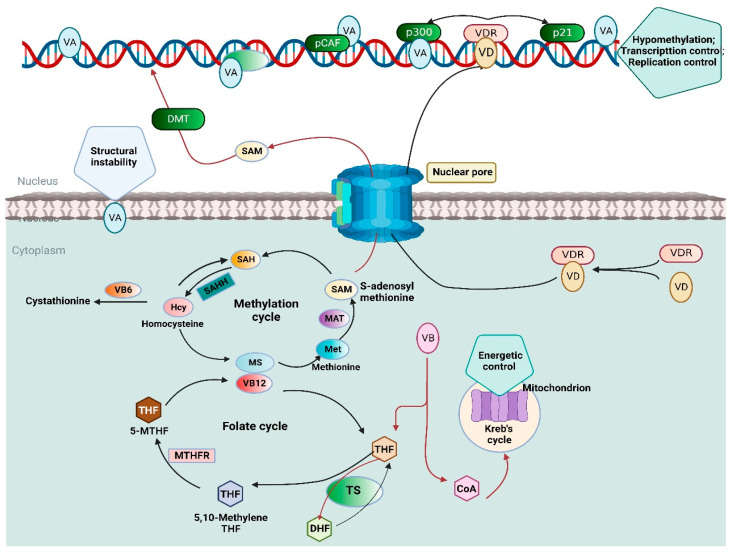
An overview of the impact of vitamin A, B complex, and D on cellular biochemistry. (Created with BioRender.com) CoA: Coenzyme A; DHF: Dihydrofolate; DMT: DNA methyltransferase; Hcy: Homocysteine; MAT: Methionine adenosyl transferase; MS: Methionine synthase; MTHFR: Methyltetrahydro-folate reductase; PCAF: p300/CBP-associated factor; SAH:S-adenosyl homocysteine; SAHH: S-adenosyl homocysteine hydrolase; THF: Tetrahydrofolate; TS: Thymidylate synthase; VDR: Vitamin D receptor.

Therefore, reliable and valid nutritional biomarkers are required to monitor nutritional status and accurately reveal the association between nutrition and disease. Numerous studies have reported a correlation between vitamin status and disease risk by employing nutritional biomarkers [45,46,47,48]. In particular, the nutritional biomarkers of vitamin A (retinol), vitamin B9 (folate), vitamin B12 (cobalamin), and vitamin D were well-established and used in epidemiological studies to depict the deficiency in health outcomes. An overview of the impact of vitamin A, B complex, and D on cellular biochemistry is shown in Figure 1.

Vitamins A, B complex, and D have a significant impact on cellular biochemistry as they modify the transcriptional landscape of the cell. Vitamin A affects cell expression through attaching to the transcription factor p300, which is involved in transcription and replication. Another method how vitamin A modifies the epigenetic levels of genetic expression is via binding to the control of histone acetyltransferase pCAF. Vitamin B is essential for cell metabolism because it is incorporated into CoA, which participates in the Krebs cycle (TCA cycle) in the mitochondrial matrix. The folate cycle begins with dietary folate being transformed into dihydrofolate (DHF), which is subsequently converted into tetrahydrofolate (THF) by the enzyme dihydrofolate reductase (DHFR). Serine hydroxymethyltransferase then converts THF to 5,10-methyleneTHF. Methylenetetra-hydrofolate reductase (MTHFR) may also convert 5,10-methylene THF to 5-methytetrahydrofolate (5-MTHF). 5-MTHF contributes a methyl group to regenerate methionine from homocysteine (Hcy), which is processed by methionine synthase (MS), which requires B12 as a cofactor in the form of methyl cobalamin. S-adenosylmethionine (SAM) is synthesized for usage by numerous methyltransferases. During the methyltransferase activities, SAM is demethylated to create S-adenosylhomocysteine (SAH), which is then hydrolyzed by S-adenosylhomocysteine hydrolase (SAHH) to form Hcy. Vitamin D is bound by vitamin D receptor (VDR), which enhances its translocation into the nucleus. The complex VDR/D attaches to certain recognition sequences termed VDREs found in the promoters of various genes, including those associated with the control of the cell cycle and the epigenetic modification.

### 3.1. Vitamin A Biomarker

Vitamin A is a vital nutrient that consists of a set of fat-soluble compounds such as retinol, retinene, and retinoic acid. It must be acquired from the food because of the incapacity of its synthesis by humans. Vitamin A is abundant in food sources such as liver and dairy products. Meanwhile, colorful fruits and vegetables are sources of provitamin A carotenoids, which can be further transformed to vitamin A in human body at a rate of 10% to 15% [49,50,51]. After digested, dietary retinol, retinyl esters and carotenoids are released from the food matrix and absorbed by small intestinal mucosal cells to form retinal, the majority of which is esterified and transferred to retinyl ester-containing chylomicrons for storage and use in the liver. The liver serves as the primary location of vitamin A storage when there is an adequate supply of vitamin A in the diet. Retinyl esters could be preserved in trace amounts in gut and renal. Retinyl esters are hydrolyzed into free retinol before being released from the liver, where it subsequently forms a complex with retinol-binding protein (RBP) before being secreted into the blood [52,53]. It has been demonstrated that vitamin A has a role in maintaining cell growth and differentiation, immune function, and vision health. Therefore, an adequate vitamin A status is critical for normal growth and development [54,55]. The gold standard for determining vitamin A levels and function is serum retinol, serum retinyl esters, RBP and breast-milk retinol, since they are closely related to liver vitamin A concentrations [20].

Vitamin A deficiency was assessed in population-level prevalence surveys. Measurement of serum retinol concentrations by high performance liquid chromatography (HPLC) is a typical method for evaluating vitamin A concentrations. The recommended threshold for serum retinol concentrations is 0.7 μmol/L, and a low circulating retinol level was reported in almost 30% of the pre-school children and 15% of pregnant women globally [56]. Serum retinol should be monitored for vitamin A deficient populations for determining supplementation strategy, while it is not reflective of vitamin A status in overall healthy populations [20]. Therefore, it is necessary to select appropriate nutritional biomarkers to evaluate vitamin A levels in the people based on the actual situation.

#### Application of Vitamin A Biomarkers

Various studies reported the role of vitamin A in disease development with a deployment of vitamin A biomarkers. Due to the immunomodulatory properties of vitamin A, insufficient vitamin A may cause an imbalance between anti-inflammatory and pro-inflammatory substances, resulting in impaired immune function in sepsis. There is scientific evidence to suggest that individuals with sepsis may experience worse clinical outcomes as a result of vitamin A deficiency (VAD). Importantly, VAD is quite common in kids, particularly in preschoolers. The association between VAD and sepsis, however, is poorly understood due to a lack of research findings. Zhang et al. investigated the link between sepsis and vitamin A deficiency. In this study, 156 children with sepsis and 49 children with impaired health were included. In a logistic model, pediatric risk of mortality scores and vitamin A levels were independently associated with septic shock. Vitamin A deficiency was prevalent in children with sepsis, and severe vitamin A deficiencies were linked to an increased risk of death [21]. A study that explored the relationship between the plasma vitamin A concentration and COVID-19 severity discovered a strong and negative correlation between plasma vitamin A levels and the levels of inflammatory markers (CRP, ferritin) that reflect the severity of severe acute respiratory syndrome coronavirus 2 (SARS-CoV-2) infection. Vitamin A is particularly beneficial in the treatment of infectious disorders, particularly lung infections. It is essential for the growth and repair of lung tissue following infection, and hence may have an impact on the recovery from serious COVID-19 pneumonia. Severe infections and inflammation can also have a deleterious impact on vitamin A status, and numerous mechanisms have been documented, including vitamin A loss in the urine, impaired hepatic mobilization of vitamin A, and decreased intestinal vitamin A absorption during infection. Furthermore, vitamin A levels in hospitalized patients were much lower than those in recovered patients, and critically ill hospitalized patients had substantially lower levels of vitamin A than moderately ill patients. Acute Respiratory Distress Syndrome (ARDS) development and mortality were significantly correlated with vitamin A plasma levels, with a cutoff at 0.7 µmol/L [57].

Vitamin A is required for the immune system to react properly to infection. Infection also affects vitamin A biomarkers, making it difficult to assess vitamin A deficiency and, hence hindering clinical interventions. For example, during an acute illness or inflammation, hepatic release of retinol and RBP is temporarily decreased, rendering vitamin A evaluation inaccurate. Therefore, appropriate adjustments for nutritional biomarkers enhance the accuracy in the prediction of nutritional status and its relationship with diseases [52]. A study on pediatric patients with acute febrile illness using a four-adjustment strategy (BRINDA regression correction, CRP-only adjustment factor, Thurnham correction factor, and proof-of-concept for a proposed interleukin 6 regression model) to measure RBP concentrations reported that no participants were classified as vitamin A deficiency (RBP < 0.70 μmol/L) and 14.0% were classified as vitamin A insufficiency (RBP < 1.05 μmol/L), which was different from the results generated by using the conventional unadjusted RBP measurement. During the acute visits, vitamin A deficiency was related with elevations in macrophage inflammatory protein 1-alpha and proinflammatory biomarkers. This study exemplified the utilization of vitamin A surveillance to monitor the health status or disease progression in children with heavy incidence of malnourished and infectious diseases [22].

### 3.2. Vitamin D Biomarker

Vitamin D can be synthesized endogenously by human body following cutaneous exposure to Ultraviolet-B radiation. However, dietary intake is commonly required to treat or prevent Vitamin D deficiency, especially among the population with insufficient sun exposure. Vitamin D insufficiency is a global health issue, and research has related it to poor survival in malignancies such as diffuse large B-cell lymphoma, chronic lymphocytic leukemia, non-Hodgkin lymphoma, and peripheral T lymphoma cell lymphoma [24]. Foods containing abundant natural vitamin D include sardines, salmon and cod liver oils, mushrooms, and liver or organ meats. There are two sorts of vitamin D: vitamin D2 (ergocalciferol) and vitamin D3 (cholecalciferol) [58]. Vitamin D is first transformed into 25-hydroxyvitamin D in the liver by the enzyme vitamin D-25-hydroxylase, and then into the active form 1,25-dihydroxyvitamin D [1,25(OH)2D] in the kidney by 25-hydroxyvitamin D-1-hydroxylase [59]. 1,25(OH)2D performs its physiological function in target tissues by attaching to the vitamin D receptor, resulting in the up- or down-regulation of a vast number of genes [60,61].

In the human body, the predominant form of vitamin D is 25(OH)D, which possesses a lengthy half-life. Since serum or plasma 25(OH)D concentration represents the contributions from both food and skin production, it is commonly acknowledged that these measurements should be utilized to assess vitamin D status. Therefore, the 25(OH)D concentration could be utilized as a biomarker to reflect the total vitamin D status [62,63]. Serum 25(OH)D can be evaluated by applying an electrochemiluminescence immunoassays, and a cutoff value of <30 nmol/L was defined as deficient, 30–50 nmol/L as insufficient, and ≥50 nmol/L as sufficient [64].

#### Application of Vitamin D Biomarkers

The correlation between serum 25(OH)D concentrations and numerous disorders in general population is poorly understood. More pertinent investigations are required to properly and quantitatively investigate the connection between 25(OH)D and the diseases. Vitamin D is thought to regulate the renin-angiotensin system (RAAS), promote pancreatic beta-cell activity, inhibit systemic inflammation, and reduce carotid intima-media thickness, meaning that it is a possibly modifiable risk factor in cardiometabolic disorders. A cross-sectional study including 1714 people from Henan province in China analyzed serum 25(OH)D and eight cardiometabolic biomarkers to completely and statistically investigate the relationship between cardiometabolic features and 25(OH)D levels. Fasting serum glucose (GLU) and low-density lipoprotein cholesterol (LDL-C) were shown to be negatively connected with 25(OH)D concentrations and positively correlated with high-density lipoprotein cholesterol (HDL-C) [65]. A meta-analysis with 20 cohort studies (*n* = 217,235) reported that when the 25(OH)D concentration was approximately at 50 nmol/L or the vitamin D consumption was around 12 μg per day, the risk of stroke was at its lowest level, compared to a higher or lower percentile. Mechanistically, activated vitamin D may prevent the formation of foam cells by decreasing the cellular uptake of cholesterol, and subsequently reduce the development of atherosclerosis and stroke. In consistent, vitamin D insufficiency is correlated with an increased incidence of secondary hyperparathyroidism [25]. In addition, Kwon et al. revealed that the bioavailable 25(OH)D was strongly and negatively associated with the susceptibility of nontuberculous mycobacterial pulmonary disease (NTM-PD) [66]. The vulnerability to NTM-PD was substantially correlated with bioavailable 25(OH)D concentrations, but not with the effectiveness of therapy. Lower 25(OH)D bioavailability may be a potential risk for NTM-PD.

Numerous epidemiological studies have demonstrated that cancer incidence and mortality rates were increased in people who had lower vitamin D levels. The pathways via which vitamin D affects cancer biology include apoptosis, angiogenesis, cell differentiation, inflammation, and immunological regulation [23,24]. To verify the association between 25(OH)D and cancer, Johnson et al. conducted a population-based trial on 8700 individuals with a mean SD 25(OH)D value of 29.7 ± 12.8 ng/mL. During a median follow-up of 4.6 years, a total of 761 people developed cancer. In comparison to the participants with a 25(OH)D value of 20–50 ng/mL, those with a 25(OH)D value of less than 12 ng/mL had an increased incidence of non-skin cancer. Lower 25(OH)D levels (12–19 ng/mL) were correlated with a higher cancer mortality, compared with the lowest percentile. Participants with 25(OH)D levels less than 12 ng/mL had a 1.6-fold increased risk of non-skin cancer compared to those with normal 25(OH)D levels, whereas those with levels below 12 ng/mL and between 12 and 19 ng/mL had 3.5- and 2.8-fold higher cancer-related death rates than the reference group, respectively [67].

To examine serum 25(OH)D insufficiency and its prognosis significance in Hodgkin lymphoma (HL) patients who have recently been diagnosed, Qin et al. included 77 patients with a median 25(OH)D level of 44.5 nmol/L and 16 of whom were classified as 25(OH)D deficient. In a multivariate Cox regression model, 25(OH)D deficiency was revealed to be an independent predictor of progression-free survival (PFS) and overall survival. ROC curves demonstrated that the International Prognostic Score (IPS) combined with 25(OH)D deficit predicted PFS and overall survival rate more accurately than the IPS alone, indicating that the 25(OH)D status might be employed as an additional biomarker for predicting HL prognosis. It has been demonstrated that 1,25(OH)2D promotes the expression of PD-L1 and PD-L2 encoding genes which suppresses T-cell mediated anti-tumor immunity [23]. In a related study that analyzed the relationship between serum 25(OH)D and mantle cell lymphoma (MCL), the researchers revealed that 25(OH)D insufficiency was an independent prognostic factor for PFS and overall survival rate. These studies indicate that the 25(OH)D concentration, in combination with MIPI-c, improved the prognostic capacity of MCL [24]. Nutritional biomarker 25(OH)D is the predominant circulating form of vitamin D, which monitoring biomarker concentrations in the body can benefit with disorder prevention and treatment.

### 3.3. Vitamin B9 (Folate) Biomarker

Vitamin B9, a nutrient that naturally occurs as folate, is an essential micronutrient required for RNA and DNA synthesis as well as cell division, growth and development [68,69,70]. Folate is essential for one-carbon metabolism, which mediates the biosynthesis of amino acids and nucleic acids [71]. Folate deficiency due to insufficient dietary consumption is related to a variety of health issues, including megaloblastic anemia, cardiovascular disease, and neural tube abnormalities (NTD) [72,73]. This occurs primarily in populations whose intake of fresh fruits, vegetables, and legumes is low and whose diets rely more heavily on processed foods. According to the Nordic Nutrition Recommendations, the average requirement for folate is 200 μg/day and the recommended intake (RI) is 300 μg/day to keep blood folate concentrations above cut-offs. RI for women in reproductive age is 400 μg/day to prevent the development of NTDs [74,75]. The primary form of vitamin B9 that is naturally present in food is folate, while folic acid is the form of the vitamin utilized in dietary supplements and food fortification/enrichment. Since the excess intake of folic acid from fortified processed food is relatively prevalent in countries with mandatory fortification, the principle of maximum intake needs to be followed to prevent adverse health outcomes from excessive intake [16]. This section investigates whether different biomarkers are reliable indicators of vitamin B9 deficiency and their impact on epidemiology studies.

As indicators of vitamin B9 status, biomarkers such as serum folate, red blood cell (RBC) folate, and plasma homocysteine concentrations were widely used in previous studies. Serum folate concentrations are strongly indicative of folic acid consumption, with the greatest amounts reported in those who take folic acid supplements or eat fortified foods. Serum folate measurement is the primary biomarker for changed folate exposure, implying previous food consumption (i.e., short-term status). RBC folate is a reliable predictor of long-term folate level since it indicates the quantity of folate that accumulates in RBCs during erythropoiesis, revealing folate status over the previous 120 days. Like serum folate, RBC folate is extremely sensitive to folic acid intervention; however, at comparable intervention doses, natural dietary folates frequently result in a lower response in RBC folate than folic acid. Plasma homocysteine testing offers a sensitive functional biomarker of folate status. Folate-mediated one-carbon metabolism is a network of three interconnected metabolic pathways that includes homocysteine remethylation to methionine. Plasma homocysteine levels are generally observed to be raised when folate levels are inadequate or insufficient. Serum and RBC folate were often measured by microbiological assays, while the plasma homocysteine concentrations were frequently employed as a non-specific “functional” predictor of folate status [16].

#### Application of Folate Biomarker

Metabolic alterations linked to insufficient folate status have been related to an increased risk of chronic disorders such as cancer, cognitive impairment, and cardiovascular disease [26,27,28]. This validates serum folate as a potential biomarker with the possibility to influence the risk of dementia. To analyze the correlations between serum folate levels and the development of dementia, Rotstein et al. recruited 27,188 health elderly people with their serum folate levels measured and followed up for 10 years. A serum folate level below 4.4 ng/mL was defined as deficiency. As a result, higher risk of dementia and all-cause death were found in people with folate deficiency compared with those with normal folate levels, indicating that the serum folate concentration may serve as a predictor for dementia, and folate supplement may be a prophylactic precaution or a part of therapeutic procedures against dementia [26]. It was postulated that a folate deficit affects homocysteine levels and impairs DNA repair in neurons, raising the risk of dementia and oxidative damage in the vascular system, which might speed up age-related malfunction and cell death.

Folate is vital in polyneuropathy such as Guillain-Barr syndrome (GBS), a diverse neuropathy characterized by acute bilateral limb paralysis following immunological responses. Gao et al. explored the correlation between the level of serum folate and the severity and progression of GBS and reported that individuals with regular folate concentrations exhibited slower disorders progression than those with lower folate concentrations. In addition, a normal folate level upon admission was an independent predictor of accelerated GBS development. However, owing to the cross-sectional design of the study, more research is required to explore the potential of establishing folate status as a predictive biomarker for GBS [27]. Additionally, folate is thought to be significantly demanded by tumor cells in terms of giving methyl groups and synthesis purines and pyrimidines. Awwad et al. measured the blood levels of folates and methylation markers such as S-adenosylhomocysteine (SAH) and S-adenosylmethionine (SAM) in the blood of males with prostate cancer (PCa) or benign prostatic hyperplasia (BPH) and portrayed a negative association between the (6S)-5-CH3-H4 folate levels and the grade of tumor malignancies. Furthermore, folate metabolism was altered in older patients with progressed PCa as well as in youthful patients with low severity PCa but lower plasma SAM levels [28].

Folate and other B-group vitamin deficiencies such as vitamin B12 insufficiency, are a prevalent health issue affecting both high- and low-income nations. Through critical physiological processes including DNA synthesis, cell division, RBC maturation, and nervous system myelination, these B-vitamins are essential for optimum growth and development. In a randomized controlled trial, Solvik et al. explored the effects of dietary intake of B-vitamins on neurodevelopment in Norwegian preschool children and measured plasma levels of folate, cobalamin, plasma total homocysteine (tHcy), and methylmalonic acid (MMA) as biomarkers of food intake. As expected, plasma folate concentrations were negatively correlated with tHcy, children who took vitamin supplements had plasma folate concentrations that were 51% greater than those who did not. A 23% decreased plasma folate levels were linked to eating red meat for supper more than twice per week [76]. As a result, this will aid kids in developing a balanced diet and preventing B vitamin deficiencies.

### 3.4. Vitamin B12 Biomarker

Vitamin B12 are enriched in animal foods such as meat, eggs, and dairy products. Upon ingestion, dietary vitamin B12 binds to haptocorrin, which protects vitamin B12 against acid while it passes through the stomach. In the gut, bound vitamin B12 is released by pancreatic enzymes and binds to intrinsic factor (IF). Vitamin B12-IF attaches to cubulin receptors in the terminal ileum, where it is absorbed by receptor-mediated endocytosis. When vitamin B12 is absorbed, it is released into the plasma, where 20–25% of it is bonded to transcobalamin (TC) (also known as holo-TC or active B12) and absorbed by cells for further usage. Another 75–80% of vitamin B12 is accumulated in the liver [77,78].

Vitamin B12 deficiency is common worldwide, especially in groups of individuals who consume less animal products due to lifestyle choices, poor socioeconomic level, or ethical considerations. The daily recommended dose of vitamin B12 is 4.0 μg, with higher requirements during pregnancy and lactation, according to the European Food Safety Authority. Children from deficient mothers and elderly people are at risk for vitamin B12 deficiency [79]. Dietary evaluation of vitamin B12 consumption can be a reliable predictor of the risk of deficiency, but biomarkers are required to evaluate the present vitamin B12 status. Four biomarkers are frequently used to reflect circulating vitamin B12 status: serum (or plasma) B12 concentration, serum holotranscobalamin (holoTC) concentration, serum methylmalonic acid (MMA) concentration, and plasma tHcy concentration [15]. HoloTC is the portion of circulating vitamin B12 that could be absorbed by cell, whereas tHcy and MMA increase with vitamin B12 shortage because vitamin B12 is required as a cofactor for the methylmalonyl-CoA mutase and methionine synthase, respectively. Wijngaarden et al. investigated the sensitivity and validity of serum vitamin B12, HoloTC, MMA, and tHcy as biomarkers of dietary vitamin B12 intake among the participants with increased tHcy concentrations (≥12 µmol/L). Interestingly, increasing B12 consumption by 2-fold was linked to a 9% rise in total serum B12, a 15% increase in HoloTC, a 9% drop in MMA, and a 2% decrease in tHcy, indicating that the changes of biomarkers were unproportional to the dietary changes. Therefore, adjustments are warranted when serum vitamin B12, HoloTC, MMA, and tHcy are measured to reflect the changes in dietary vitamin B12 [80].

#### Application of Vitamin B12 Biomarker

Vegans are more susceptible to vitamin B12 and other nutritional deficiencies. However, the short-term consequences of shifting to a vegan diet on the metabolism of vitamin B12 are poorly understood. Serum vitamin B12 and holotranscobalamin, two systemic indicators of vitamin B12 status, may react significantly to a reduction in vitamin B12 consumption. Lederer et al. conducted a four-week trial in which 53 healthy volunteers were randomly allocated to either a controlled unsupplemented vegan diet or a meat-rich diet (MD). Serum concentrations of vitamin B12, holo-transcobalamin (holo-TC), methylmalonic acid (MMA), and total plasma homocysteine (tHcy) were measured to evaluate the status of vitamin B12. After 4 weeks of intervention, Holo-TC levels in the vegan diet group were considerably lower than in the MD group, although MMA and tHcy levels were unaffected. After 4 weeks, vegan diet group had decreased vitamin B12 consumption, which was reflected in lower blood vitamin B12 concentrations and lower holo-TC. As a result, plasma holo-TC might be a reliable and sensitive biomarker for assessing vitamin B12 status in vegans [81].

Micronutrient deficiencies, such as vitamin B12 deficiency, are increased after bariatric surgery, because of the significant changes in anatomy and physiology of the gastrointestinal tract. Kornerup et al. investigated early alterations in B12 status biomarkers after bariatric surgery. Blood was collected before surgery, as well as two and six months afterwards, to determine the vitamin B12, holoTC, and MMA concentrations. Although the decline in serum vitamin B12 was not significant until 6 months after surgery, a substantial decline in plasma holoTC and a rise in MMA were observed at 2- month post-surgery. After six months following surgery, these changes were more obvious, indicating that the impairment of postoperative vitamin B12 absorption capacity is time dependent. Thus, HoloTC and MMA may outperform serum vitamin B12 as nutritional biomarkers of vitamin B12 status among patients who went through bariatric surgery. These findings challenge the conventional belief that the circulating vitamin B12 concentration is stable with the hepatic vitamin B12 supply. Instead, vitamin B12 supplementation is required after surgery [29]. S-adenosylmethionine, which has antidepressant capabilities and is necessary for folate and B12 synthesis, may be involved in the link between vitamin B12 and depression. Additionally, vitamin B12 level, together with other B-vitamins such as folate, may be negatively related to the incidence of depression. Nevertheless, it is pivotal to realize that a lower folate and VB12 status was linked to poor eating habits, which indicates that dietary pattern, instead of single nutrients, may exhibit more potent effects in the development of depression [30,82,83]. B12 cofactors are crucial for the proper metabolism and function of many physiological systems. Therefore, effective nutritional biomarkers will be essential for epidemiological studies of diseases such as megaloblastic anemia, neuropathy, and dementia.

## 4. Mineral Biomarker

Minerals are dietary supplements that are essential for preserving healthy physiology and function. Mineral elements such as iron (Fe), zinc (Zn), iodine (I), and selenium (Se) are highly valued in modern healthy diets as they have special roles in cellular metabolism [84]. In addition, the oxidative or antioxidant properties of certain metals may affect cardiovascular health, and reduce the risk of anemia, cancer and so on. For instance, iron deficiency (ID) is a substantial contributor to the risk of heart disease and affects overall mortality [85]. Therefore, adequate intake of essential minerals through diet and/or supplements is recommended to promote health [86,87]. Current mineral nutrient biomarkers and their applications in epidemiology are reviewed below.

### 4.1. Iodine Biomarker

Iodine is a crucial trace element participated in the generation of the thyroid hormones triiodothyronine (T3) and thyroxine (T4) [88]. According to the recommendations of WHO, most countries set the daily iodine intake for adult at 150 μg and increase it to 250 μg during pregnancy for its critical involvement in the growth of the embryo’s nervous system [89,90]. The frequency of iodine deficient disorders has decreased over the past three decades owing to international efforts and initiatives including “salt” iodization [91]. However, iodine deficiency is still common in some few places, though. Regularly monitoring the population’s iodine status will be critical in maintaining appropriate intakes and the absence of excessive intakes. The recommended biomarkers, based on the current evaluation system, include urine iodine concentration (UIC), thyroid-stimulating hormone (TSH), thyroglobulin (Tg), T4 and T3, and goiter. These biomarkers may directly reflect dietary iodine consumption or may be linked to thyroid hormones metabolism, which can accurately reflect the body’s iodine levels [19].

**Figure 2:**
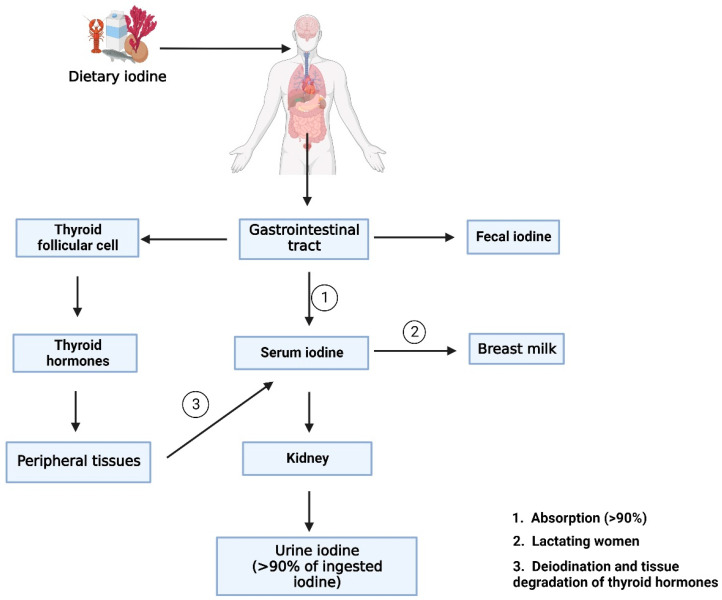
An overview of the iodine biomarkers and related metabolism pathway. (Created with BioRender.com).

In general, the population median of the UIC survey is based on a single spot sample, while causes an inaccurate assessing of iodine status. According to Bertinato’s study, iodine intake distribution based on two UIC samples, combined with an estimated mean requirement or tolerable higher intake level, cut-point method is developed and proved to be a promising approach that could be employed in population iodine status monitoring [92]. Cui et al. examined the effectiveness of serum iodine to assess iodine consumption in children, finding that serum iodine level was favorably linked with daily iodine consumption [93]. In Hlucny’s study, iodine levels were examined following a 3-day iodine-titrated diet for 10 participants. Serum iodine, Tg, and 24-hour UIC levels were measured after each diet. At baseline, a 24-hour UIC and an iodine-specific food frequency questionnaire (FFQ) were completed. UIC elevated on average by 19.3 µg/L for every gram of iodized salt ingested. These findings imply that serum iodine and 24 h UIC can represent a person’s iodine level and act as iodine status biomarkers [94]. Further research and development of more sensitive and specific biomarkers to assess individual iodine status for routine patient care is highly needed. The WHO recommends that a median UIC of more than 100 μg/L to determinate population iodine sufficiency, but some authorities suggest 60–70 μg/L [19]. The current opinion is that urine iodine concentration (UIC) is a proven biomarker for determining the iodine status of the population and will facilitate the ability to continuously monitor in nutrition programs [92,95]. An overview of the iodine biomarkers and related metabolism pathway are shown in Figure 2.

After ingestion, dietary iodine is digested by the gastrointestinal tract, and the majority of the iodine taken into the body is dispersed in the blood as serum iodine. Some of the serum iodine is converted to breast milk in lactating mothers. The other part of the iodine absorbed in the body is involved in the synthesis of thyroid hormones. After entering the thyroid follicular cell, iodine incorporated into thyroglobulin to participate in the synthesis of thyroid hormones. Then thyroid hormone is deiodination and degradation by peripheral tissues and converted back into serum iodine. The portions of serum iodine that are not absorbed and utilized by the human body are transformed into urine iodine and eliminated from the body via kidney metabolism. The remaining unabsorbed iodine is excreted to the body as fecal iodine. Serum iodine, thyroid hormone, fecal iodine, and urinary iodine produced during these metabolic processes are useful biomarkers for measuring iodine levels in the body.

#### Application of Iodine Biomarker 

During pregnancy, dietary requirements for iodine increase by 50% to 250 µg/d due to the increased production of thyroid hormones required by the mother and her fetus [35], low thyroid hormone levels may result in a variety of negative effects, notably on brain growth and development, making it worth considering specific assessment for pregnant women iodine status. Postulated pathways for how iodine shortage affects growth may involve decreased levels of insulin-like growth factor 1 (IGF-1) and IGF binding protein 3 (IGFBP-3). Kanike et al. evaluated urine iodine concentrations and thyroid function in 50 mothers and infants at birth, one week, one, two, three, and four months, as well as close discharge. Their findings revealed that iodine deficit was prevalent in pregnancies and neonates born at exceptionally low gestational age compared to full term, particularly in pregnant mothers at risk of hypothyroidism. To minimize iodine shortage, iodine supplementation should be explored throughout pregnancy and postpartum [96].

Placental concentrations may better represent the pregnancy’s long-term iodine status. Gestational diabetes mellitus (GDM) is a disorder that causes glucose intolerance throughout pregnancy and subsequently subsides after birth. Adult’s iodine deficiency has been associated with altered insulin and glucose homeostasis, primarily through thyroid hormones. To investigate the correlation between placental iodine concentrations and diabetes risk in pregnant women. A study evaluated the prevalence of gestational diabetes mellitus (GDM) in 471 mother-infant pairs in a birth cohort of 24 to 28 weeks gestation. Neven et al. measured iodine concentrations in placenta, insulin levels in maternal and cord blood, and the Homeostasis Model Assessment (HOMA) for insulin resistance (IR) index and β-cell activity to investigate the association between placenta iodine concentration and GDM. Higher placental iodine levels were shown to reduce the incidence of GDM. Plasma insulin concentrations in cord blood were shown to be inversely linked to placental iodine concentration. Furthermore, changes in plasma insulin level, HOMA-IR score, and β-cell activity were connected to reduced placental iodine load. These results support the hypothesis that mild to moderate iodine deficiency in healthy pregnant women is related to subclinical and early-onset alterations in normal insulin homeostasis [34].

### 4.2. Iron Biomarker

Iron is a crucial trace element for human health as it regulates a broad range of biological reactions. Many metabolic processes, including energy generation, DNA synthesis, and oxygen transport, are depending on iron’s ability to flip between ferric and ferrous forms. Red meat, green leafy vegetables, nuts, and fortified morning cereals are among foods with a reasonably high iron content; however, the absorption of iron is quite varied. As the most prevalent micronutrient deficit, iron deficiency affects around one-third of the population. Anemia brought on by iron deficiency owing to malnutrition affects roughly 30% of non-pregnant women, 40% of pregnant women, and children under the age of five [97]. Plasma iron levels are regulated by the hepcidin/ferroportin system, and iron enters the circulation via ferroproteins for transportation to the liver and bone marrow, respectively, for red blood cell production and storage. Systemic iron levels are closely regulated since the body has no other way to eliminate iron than through blood loss or cell turnover [98].

**Figure 3:**
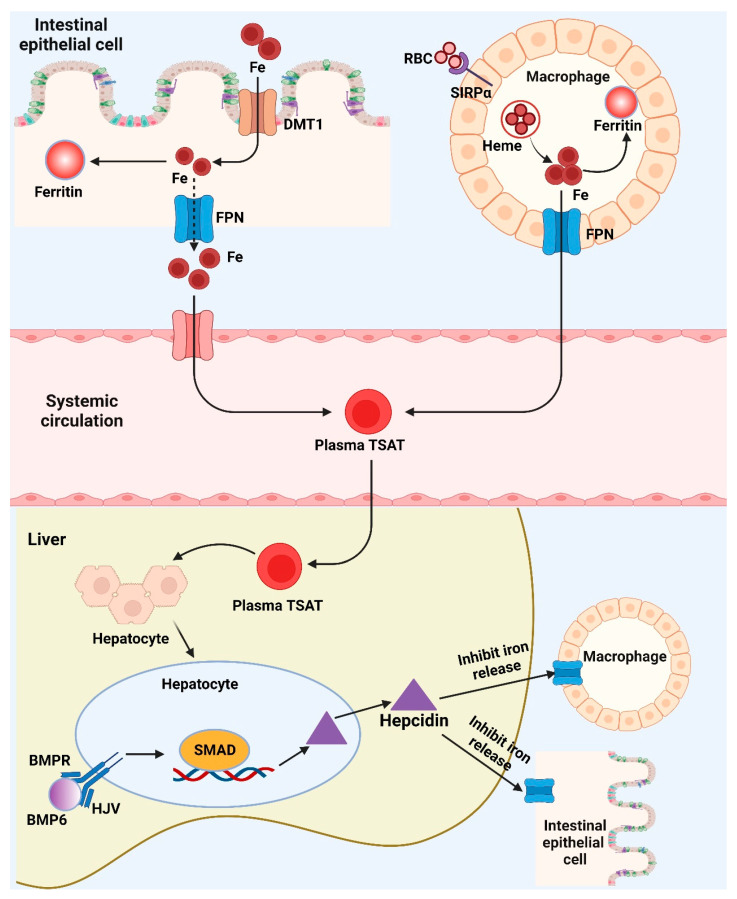
Brief depiction of the systemic iron metabolism and potential iron biomarkers. (Created with BioRender.com). BMP6: Bone morphogenetic protein 6; BMPR: Bone morphogenetic protein receptor; DMT1: Divalent metal transporter 1; Fe: Iron/Ferrum; FPN: Ferroportin; HJV: Hemojuvelin; RBC: Red blood cell; SIRPα: Signal regulatory protein alpha; SMAD: Small-body-size mothers against decapentaplegic homolog 1; TSAT: Percentage transferrin saturation.

As iron depletion progresses, declines in serum ferritin levels are correlated with drops in bodily iron storage. Since the onset of iron-deficient erythropoiesis, body iron stores are no longer sufficient to produce the quantities of iron necessary for the generation of hemoglobin and other functioning iron compounds. In the US National Health and Nutrition Examination Survey, circulating hemoglobin concentrations and soluble transferrin receptor (sTfR) concentrations were investigated as two physiological indicators of the beginning of iron-deficient erythropoiesis. When iron-deficient erythropoiesis occurs in an otherwise healthy person, the amount of accessible iron is inadequate to enable hemoglobin synthesis, resulting in a reduction in hemoglobin concentration. Independent of hemoglobin concentration, sTfR concentration is a sign of iron-deficient erythropoiesis. When iron-deficient erythropoiesis occurs, erythroid progenitors and other iron-requiring cells in the body increase the expression of cellular transferrin receptor 1 to acquire more iron. An extracellular portion of the cellular transferrin receptor called plasma sTfR, which is shed into the plasma and serves as a gauge for the mass of the cellular transferrin receptor. The erythroid marrow is where around 80% of plasma sTfR is generated, and when there is no iron deficiency, the concentrations are related to the volume of the erythroid marrow. Plasma sTfR concentrations are proportional to cellular iron demand in the absence of erythroid hyperplasia and are a sensitive, quantitative biomarker of the early stage of iron insufficiency: iron-deficient erythropoiesis. A physiological foundation for determining the onset of iron-deficient erythropoiesis is the serum ferritin level at which hemoglobin level starts to fall and sTfR commences to increase. Systemic iron metabolism and potential iron biomarkers are shown in Figure 3. Nutritional iron biomarkers can be measured in circulating red blood cell mass, serum ferritin (SF), percentage transferrin saturation (TSAT), RBC protoporphyrin and serum (soluble, plasma) transferrin receptor (sTfR), and hemoglobin. Among these biomarkers, hemoglobin is the most widely utilized indicator for iron deficiency diagnosis, however it is not sensitive or specific. Currently, SF is more often used as an indicator of iron storage and deficiency risk, although some studies suggest excluding inflammation status [18].

The iron transporter DMT1 transfers dietary iron over the border membrane and into the enterocyte. After entering the cell, the iron has two possible storage locations: the intracellular iron storage protein-ferritin or the iron export protein-ferropotin, which allows the iron to leave the cell and enter the circulatory system. In the meantime, macrophages recognize RBCs via SIRPα, recycle RBC iron, which may subsequently be transformed into heme and exported by FPN. BMP6 will bind to the BMP receptor and its co-receptor HJV, activating SMAD signaling and promoting the transcription of hepcidin when the body’s iron stores are suitably loaded. The liver produces hepcidin, which binds to FPN on macrophages and enterocytes and stimulates its degradation. This inhibits the release of iron from macrophages and the intestines into the circulation. Fe, ferritin, and plasma TSAT can serve as useful biomarkers for iron status by reflecting the body’s iron levels in numerous metabolic pathways.

#### Application of Iron Biomarker

Children and women are specific populations who require efficient biomarkers to monitor their iron states. There is evidence showed that decreased serum ferritin concentrations resulted in a decrease in median hemoglobin concentration, while sTfR concentration rose, with both changing in a curvilinear pattern in children and non-pregnant women. The relationship between two independent iron-deficiency indicators, hemoglobin and sTfR, provided a threshold for serum ferritin concentrations of around 20 ng/mL in children and 25 ng/mL in non-pregnant women, which may be more clinically and epidemiologically relevant [99].

It’s interesting to note that iron influences myocardial exercise tolerance and cardiac performance in addition to anemia. Iron also plays an important part in the metabolism of the heart and skeletal muscles through mitochondrial activity. Chronic heart failure (HF) is frequently associated with iron deficiency, which can be detected from blood test results. Ueda et al. investigated the impact of serum iron levels at discharge on the prognosis of 615 patients admitted to the hospital with acute decompensated heart failure (ADHF). According to their findings, the complication rate was much greater in the low iron group. Low serum iron levels could be an independent predictor of the composite outcome after controlling for confounders such as hemoglobin and ferritin concentrations, indicating that iron may play a role in the pathogenesis of ADHF through non-hematopoietic activities [32]. Gabriele et al. detected different definitions of iron deficiency, finding that patients with TSAT <20% and serum iron ≤13 mmol/L resulting higher mortality which was independent of HF phenotype [31].

Since iron plays a crucial role in physiological activities, sports population are particularly susceptible to iron disorder. Among adolescent female athletes, 52% is considered iron deficiency according to a related study. Zhang et al. comprehensively interpret the iron status of Chinese athletes using a multi-index evaluation system and proposed specific Chinese athlete thresholds of SF for 12 ng/mL in female and 30 ng/mL in male [100].

### 4.3. Zinc Biomarker

As a dietary essential trace element, zinc plays a key role in several metabolic processes as an intracellular metal [101,102], especially at the beginning of life. Zinc deficit is a serious health problem in many regions because of inadequate dietary intake, particularly in young children. Lack of zinc can damage the immune system and increase the prevalence of infectious disorders such as pneumonia, malaria, and diarrhea worldwide [103]. It also results in a variety of non-specific general alterations in metabolism and function, such as delayed development, an increase in infections, and appearance of skin lesions [104].

**Figure 4:**
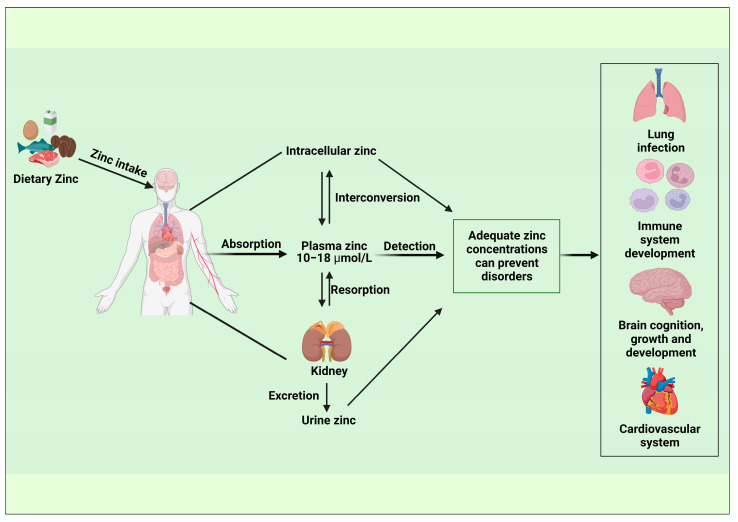
Epidemiological application of biomarkers associated with zinc homeostasis. (Created with BioRender.com).

It is challenging to identify whether there is an excess or deficiency of zinc since cellular, tissue, and whole-body zinc homeostasis are tightly controlled to sustain metabolic processes across a wide range of intakes. The BOND Zinc Expert Panel suggests three parameters for evaluating zinc levels: dietary zinc intake, plasma zinc concentration (PZC), and height for age of growing babies and children [17]. Free zinc is the exchangeable and bioactive form of zinc found in serum. Multiple expert committees have approved plasma zinc concentrations (PZCs) or serum zinc concentrations (SZCs) as reliable indicators of zinc status and are used to estimate the risk of insufficient zinc in the population, even though zinc found in blood accounts for only one percent of total zinc in the body. Studies found PZC fluctuate in response to changes in the overall body’s zinc balance as well as clinical symptoms of zinc deficit, it also responded to severe dietary zinc restriction and zinc supplementation. In addition, urinary zinc is also proposed to be a good biomarker of increases in zinc exposure, but not sensitive for dietary zinc lacking unless acute lack of dietary zinc intake (<1 mg/day) [105]. These biomarkers are related to zinc homeostasis can well reflect zinc concentrations in human body, which can be applied in epidemiological studies (Figure 4).

#### Application of Zinc Biomarker

Nutritional anemia, a kind of anemia, is particularly prevalent in children and pregnant women since the synthesis of hemoglobin is associated with not only but a range of micronutrients, including iron, folate and vitamin B12. However, zinc deficiency, which is a typical symptom in children, pregnant women and patients with chronic diseases, may also lead to the development of anemia and therefore should be taken into consideration. It is typical for children, older people and chronically ill individuals to have zinc deficiency. Atasoy et al. investigated 349 primary school students aged 6.5 to 14.8 years in a case-control study. Using the receiver operating curve, the cutoff value of serum zinc level for the prediction of anemia was determined to be 71.5 μg/dL. Zinc levels were strongly correlated with hemoglobin levels, which showed that low zinc levels were primarily responsible for the observed anemia in youngsters [106].

Moreover, zinc is essential for both innate and adaptive antiviral immunity, and the antiviral response’s underlying mechanisms have been revealed to be virus specific. A study by Marina et al. showed an association between serum zinc levels and COVID-19 outcomes. Serum zinc concentrations below 50 µg/dL on admission were linked to worse clinical outcomes and increased mortality, and low SZC was a risk factor for COVID-19 outcome. Randomized clinical trials are therefore encouraged to investigate zinc supplementation as a potential method of preventing and treating people at risk of zinc deficiency [36]. Another study used a fluorescence micro-assay to examine free zinc levels in serum samples from COVID-19 survivors and non-survivors. Parallel to the previously observed total serum zinc deficit evaluated by total reflection X-ray fluorescence, free serum zinc in COVID-19 patients was much lower than in controls, while free zinc levels in survivors were significantly higher than in non-survivors. Compared with female patients, free serum zinc levels were especially low in males. This is noteworthy since being male have been identified as a risk factor for serious COVID-19. Overall, the findings indicate that lower serum free zinc levels are correlated with an increased possibility of mortality from COVID-19, implying that free zinc may be a novel prognostic biomarker for the serious and course of COVID-19 [107].

Furthermore, zinc plays crucial parts in the hepatic lipid metabolism as well as scavenging free radical oxygen species in humans. For example, in the liver, zinc functions as a prominent activator of autophagy mediated lipophagy, which lowers lipid accumulation and stimulates lipolysis. Kim et al. recruited 300 patients with nonalcoholic fatty liver disease (NAFLD) for the investigation of the relationship between serum zinc level and the NAFLD. The results showed that the mean serum zinc concentration was 139.8 ± 29.9 μg/dL and the fibrosis-4 index increased substantially with decreasing serum zinc levels. On multivariate analysis, severe liver fibrosis in NAFLD was linked to diabetes, male gender, and zinc levels <140 μg/dL. Low serum zinc levels are independent risk factors for severe liver fibrosis in NAFLD [37].

## 5. Phytonutrient Biomarker

Phytonutrients are non-essential nutrients present in natural plants that are beneficial to human health. The phytonutrients contained in each plant are different. Among them, important phytonutrients include polyphenols, carotenoids, anthocyanins, chlorogenic acid and curcumin, which can regulate the physiological functions of the human body [108]. For example, tea polyphenols contain potent antioxidant properties, promote the immune system’s performance, and support the improvement of physical health [109]. Consuming phytonutrients through the diet may improve health and prevent chronic degenerative conditions including cancer, cardiovascular, and neurological illnesses [110,111]. Biomarkers are a type of useful tools for determining the bioavailability of phytonutrients in humans, and their concentration in plasma, serum, or urine is the major indicator of a phytonutrient’s status. There are also emerging biomarkers from recent work in this field, such as stool polyphenols, breastmilk carotenoids, or macular pigment density for lutein and zeaxanthin [112,113]. Research evidence based on biomarkers and epidemiological studies can effectively guide nutritional supplementation.

### 5.1. Biomarker of Polyphenol and Its Application

Dietary polyphenols are a varied family of 500 distinct compounds with a wide range of structural variations. Numerous scientific studies have shown that dietary polyphenols help protect against chronic illnesses including cancer, diabetes, and cardiovascular disease [114]. A mean daily consumption of 850 mg of total polyphenols was reported [115]. Recent advances in analytical technologies and metabolomics have enabled massive collections of polyphenols in the blood or urine to be assessed as indicators of polyphenol metabolite exposure. Establishing a connection between biomarkers of dietary components and epidemiological research will aid in the provision of polyphenol supplements to persons who require them.

Cardiovascular diseases are the primary reason for mortality globally, and numerous studies have shown the preventive advantages of polyphenols on the cardiovascular system [108]. The cardiovascular protective effects of polyphenols can be linked to a variety of pathways, including anti-inflammatory activities, antioxidant capacity, platelet aggregation inhibition, and antithrombotic properties [110]. In a longitudinal research, 573 subjects were divided into three groups and their total polyphenol excretion (TPE) levels were examined. To help with solid phase extraction, Folin-Ciocalteu was employed to assess TPE in urine samples. Utilizing multiple linear regression models, the relationships between clinical cardiovascular risk variables and TPE were examined. During a 5-year follow-up, there were notable negative associations between changes in TPE and plasma triglyceride levels, glucose levels, and diastolic blood pressure. According to the results, increasing the consumption of polyphenols—measured as TPE in urine—might have a protective effect against several cardiovascular risk factors [112].

An expanding amount of epidemiological evidence suggests that individuals who consume a diet high in fruits and vegetables have a reduced probability of contracting chronic diseases as well as a lower overall mortality [110,111]. To explore the relation between total urinary polyphenols (TUP) and total dietary polyphenols (TDP) with cognitive decline in elderly population. Rabassa et al. recruited 652 elderly individuals without dementia and performed a 3-year follow-up test to explore the association between polyphenol intake and cognitive ability, using both the Mini Mental State Examination (MMSE) and the Trail-Making Test (TMT) to evaluate cognition. Higher TUP levels were linked with a decreased possibility of significant cognitive decline on the MMSE and TMT in logistic regression models adjusted for baseline cognitive scores and potential covariates. In a 3-year trial, high levels of polyphenols, a dietary biomarker of polyphenol consumption, were linked to a reduced risk of significant cognitive decline in elderly populations, suggesting a protective effect against cognitive impairment [38]. Another research investigated the connection between TUP and TDP and overall mortality in older people over 12-year period, in which 274 individuals died throughout the follow-up. TUP excretion, after adjusting for age and sex, appeared to be higher in individuals who lived than those people who passed away at enrolment. As a biomarker of the aged people death prediction, the elder people with higher TUP excretion rates likely live longer than those with lower ones [116].

Furthermore, the concentration of polyphenols in the plasma may be linked to specific malignancies. Murphy et al. utilized high pressure liquid chromatography combined with tandem mass spectrometry to evaluate the plasma concentrations of 35 polyphenols in 809 colon cancer patients and 809 matched controls. Equol and homovanillic acid were linked to colon cancer risk in false discovery rate-adjusted and continuous log-transformed multivariate models. Equol concentrations were revealed to be inversely connected to colon cancer risk when comparing the extreme quintiles, but homovanillic acid concentrations were discovered to be favorably related to colon cancer occurrence. Higher homovanillic acid concentrations were linked to a higher risk of colon cancer whereas higher equol concentrations were linked to a lower risk, suggesting that some polyphenols may play a role in colon carcinogenesis [39].

### 5.2. Biomarker of Carotenoid and Its Application

Carotenoids are fat-soluble chemicals of natural plants that humans cannot directly synthesize. Numerous studies have been focused on the biological beneficial effects of carotenoids against chronic diseases [117]. A vast variety of carotenoids may be absorbed by humans, and many of them can be identified in serum and tissues. Circulating carotenoid concentrations serve as trustworthy indicators of carotenoid consumption from food and can be utilized to support nutritional evaluation methods. Moreover, compared with self-reported data, plasma carotenoid concentrations have been used as a more accurate biomarker for assessing fruit and vegetable (FV) consumption, as nearly 90% of carotenoid daily intake is provided by FV [118].

It is possible to utilize the concentration of circulating carotenoids as a reliable biomarker of carotenoid intake from diet and to complement nutritional assessment strategies. Allore et al. investigated the association of fasting plasma carotenoid concentrations with physical and metabolic characteristics after intervention. The results showed that higher body weight and waist circumference were associated with lower plasma total carotenoid concentrations, whereas higher plasma LDL and HDL cholesterol concentrations were associated with higher plasma total carotenoid concentrations. Despite having much lower dietary carotenoid intake than men, women exhibited significantly higher plasma total carotenoid concentrations. By correcting circulating carotenoid concentrations for plasma HDL-cholesterol, gender inequalities in plasma carotenoid concentrations were eliminated. When employing plasma carotenoids as indicators of dietary intake in male and female, certain physical traits and plasma lipids should be considered since they correlate with circulating carotenoid concentrations [119].

A cross-sectional study in Singapore recruited 103 middle-aged and older people to investigate the use of skin carotenoid status (SCS) and plasma carotenoids to assess FV and carotenoid consumption in the elderly. Dietary carotenoids and FV and plasma carotenoid concentrations and SCS were detected using 3-day food recordings, HPLC, and a bio-photonic scanner utilizing Roman spectroscopy, respectively. After adjusting for statistically defined sociodemographic covariates, total dietary carotenoids were positively correlated with plasma carotenoids and SCS. This indicates that these nutritional parameters may influence carotenoid cycle and skin deposition [40]. Moreover, the correlation between serum carotenoids and their intake may be weakened by hyperglycemia in type 1 diabetes with increased oxidative stress, and thus depleted serum carotenoids. In order to investigate the association between the concentrations of serum carotenoid and type 1 diabetes. Namrata et al. analyzed data from a nutritional intervention study including youngsters with type 1 diabetes. Regression analysis of the serum carotenoid measurement can be useful to estimate the conditions of glycemic control, oxidative stress, FV and carotenoid intake since these conditions were correlated with the serum carotenoid concentration. People intaking more fruit, vegetables or other carotenoid containing food likely have higher concentration of serum carotenoid, and higher hyperglycemia was associated with lower serum carotenoids. The findings of this investigation validate serum carotenoids as indicators of FV as well as carotenoid consumption in young people with type 1 diabetes [41].

## 6. Limitations of Current Nutritional Research

By reviewing the above-mentioned different types of nutritional biomarkers and their applications in epidemiological studies, we illuminated the characteristics of biomarkers from multiple aspects. Studies on nutritional biomarkers were collected through the databases PubMed, Web of science, Medline and Scopus by searching the keywords: “nutritional biomarker”, “epidemiology”, “vitamin biomarker”, “vitamin A”, “folate”, “vitamin B12”, “vitamin D”, “mineral biomarker”, “iodine biomarker”, “iron biomarker”, “zinc biomarkers”, and “phytonutrient biomarkers,” “polyphenol biomarkers,” “carotenoid biomarkers”. The properties of nutritional biomarker are analyzed and summarized by reviewing these relevant literatures. Despite the widespread usage of the current nutritional biomarkers, there are still number of issues remaining unresolved. The development of nutritional biomarkers will be constrained by these limitations. First, current nutritional biomarkers may not reflect the body’s requirements for certain micronutrients well, resulting in suboptimal consistency of biomarker levels with dietary intake levels. For example, deficiency of the mineral nutrients zinc and copper are both associated with higher concentrations of tumor necrosis factor (TNF)-alfa and interleukin (IL) -1 beta, these indicators can be used to evaluate health status or diseases risk, however, more sensitive and specific functional biomarkers are needed to be developed [17]. Multiple biomarkers are frequently combined in evaluations or novel nutritional biomarkers are developed to improve sensitivity and accuracy. Furthermore, despite some studies and reviews have been reported in this field [120], more related cohort studies and attentions are needed to promote the utilization of nutrients in disease prevention and treatment.

## 7. Conclusions and Prospective

Nutritional biomarkers are important tools that can objectively measure the individuals’ nutritional status. This review presents an overview of nutritional biomarkers for certain vitamins, minerals, phytonutrients, as well as the application of these biomarkers in epidemiology studies. Certain nutritional biomarkers may potentially serve as clinical biomarkers since they are highly correlated with specific health outcomes or prognosis. We should select appropriate biomarkers and establish a standard monitoring process for specific populations, such as pregnant women, infants, and the elderly, to provide guidelines for dietary supplement consumption. As an advanced chemical profiling method, metabolomics is increasingly used to discover new biomarkers that may integrate diet and nutrition in complex bio-systems. In the field of personalized nutrition, where the conventional “one-size-fits-all” was replaced by tailored lifestyle interventions, biomarkers are the key features in the development of predictive models and neural networks that require the feed of big data [121,122,123]. Although the establishment of a full array of biomarkers that can accurately define the cumulative dietary and nutritional exposures is far from completed, progress in this area has allowed the generation of multiple predictive models that target different disease statuses, such as obesity and diabetes. However, a standardized protocol of metabolomics, targeted or untargeted, is warranted to empower quality assurance, inter-study comparability, and data translation.

## Figures and Tables

**Table 1:** An overview of nutritional biomarkers and related epidemiology studies.

Nutritional Biomarker	Sample Type	Potential Mechanism	Related Epidemiology Studies	Ref.
Vitamin A	Serum	Pro-inflammatory and anti-inflammatory factor imbalances and reduced immunological function in sepsis may result from VA insufficiency.	Sepsis	[21]
Retinol binding protein	Blood	Acute phase response, induced by acute infection, are responsible for reductions in circulating vitamin A.	Acute febrile illness	[22]
Vitamin D [25-(OH)D]	Serum	Stimulated the expression of PD-L1 and PD-L2, suppressing T-cell mediated anti-tumor immunity	Mantle cell lymphoma/Hodgkin lymphoma	[23,24]
Vitamin D [25-(OH)D]	Serum	Activated vitamin D is an inhibitor of the renin angiotensin system (RAS), an important pathway in the regulation of the cardiovascular system.	Stroke	[25]
Folate	Serum	Folate deficiency affects homocysteine level and affects impaired DNA repair in neurons, sensitizing them to oxidative damage,	Dementia and all-cause mortality	[26]
Folate	Serum	Diminish human immune functions by affecting T and B cell differentials and the proliferation response of lymphocytes/Affects the synthesis of methyl groups and DNA in growing cells	Guillain-Barre syndrome, Prostate cancer	[27,28]
Vitamin B12	Plasma/Serum	Rearrangement compromises vitamin B12 absorption	Bariatric surgery	[29]
Vitamin B12 and Folate	Serum	Prevent impaired immune function and inflammation	Depression	[30]
Iron	Serum	Increasing hepcidin activity by inflammation in Heart Failure may lead to the development of functional Iron deficiency	Heart failure	[31,32]
Ferritin/Creatinine	Urine	Inflammation can stimulate the proliferation and activation of monocyte phagocytic system, which leads to the increase of iron absorption and retention in the reticuloendothelial system.	Lupus nephritis	[33]
Iodine	Urine/serum	Altered thyroid hormone homeostasis and dysregulation of glucose metabolism	Gestational diabetes mellitus	[34]
Iodine	Urine/breast milk	Maintaining thyroid function and body metabolism	Neurodevelopment/Iodine deficiency disorders	[35]
Zinc	Serum/Plasma	Effect on the immune system, as both the adaptive and the innate immunity, are affected by zinc levels.	COVID-19	[36]
Zinc	Serum	Inhibitor of NADPH oxidase, Zn acts as a potent promoter of autophagy-mediated lipophagy in the liver, reducing lipid accumulation and stimulating lipolysis	Hepatic fibrosis	[37]
Polyphenols	Urine/serum	Polyphenols reduce neuronal damage and death from oxidative reactions by inhibiting the generation of reactive oxygen species, lipid peroxidation, apoptosis, protein oxidation, metal chelation, and damage to cellular signaling.	Cognition disorder	[38]
Polyphenols	Plasma	Antioxidants, anti-inflammatory and estrogenic effects	Colon cancer	[39]
Carotenoids	Skin tissue/Plasma	Less susceptible to fluctuations in response to recent dietary intake and potential carotenoid degradation by heat, light and oxygen.	Metabolism disorder/Obesity	[40]
Carotenoids	Serum	Oxidative stress can deplete serum carotenoids, and thus attenuate the association of serum carotenoids with intake.	Type 1 diabetes	[41]

## Data Availability

Not applicable.
